# Predictive Role of Functional Visceral Fat Activity Assessed by Preoperative F-18 FDG PET/CT for Regional Lymph Node or Distant Metastasis in Patients with Colorectal Cancer

**DOI:** 10.1371/journal.pone.0148776

**Published:** 2016-02-10

**Authors:** Kisoo Pahk, Seunghong Rhee, Sungeun Kim, Jae Gol Choe

**Affiliations:** Department of Nuclear Medicine, College of Medicine, Korea University, Seoul, Korea; Kyungpook National University School of Medicine and Hospital, REPUBLIC OF KOREA

## Abstract

**Objectives:**

To investigate the role of functional visceral fat activity assessed by preoperative F-18 fluorodeoxyglucose positron emission tomography/computed tomography (^18^F-FDG PET/CT) in colorectal cancer (CRC) for predicting regional lymph node (LN) or distant metastasis.

**Method:**

We evaluated 131 patients with newly diagnosed CRC. They all underwent pre-operative ^18^F-FDG PET/CT and surgery. Functional fat activity was measured by maximum standardized uptake value (SUVmax) using ^18^F-FDG PET/CT. Functional visceral fat activity was measured by SUVmax of visceral fat/SUVmax of subcutaneous fat (V/S) ratio. Mann-Whitney *U* test, χ^2^ test, Fisher’s exact test, receiver-operating characteristic (ROC) analysis, Spearrman’s correlation coefficient, and uni- and multivariate logistic regression statistical analyses were done.

**Results:**

Patients with higher V/S ratio displayed a significantly higher rate of regional LN (*p =* 0.004) and distant metastasis (*p<*0.001). In addition, V/S ratio was the only factor that was significantly associated with distant metastasis. An optimal cut-off V/S ratio of 1.88 was proposed for predicting distant metastasis with a sensitivity of 84.6% and specificity of 78.8% (area under the curve: 0.86; *p*<0.0001)

**Conclusion:**

Functional visceral fat activity is significantly associated with distant metastasis in CRC patients. Furthermore, V/S ratio can be useful as a complementary factor in predicting distant metastasis.

## Introduction

Colorectal cancer (CRC) is the one of the leading causes of cancer death worldwide [1]. Regional lymph node (LN) and distant metastases are important prognostic factors of CRC [2].

Visceral obesity increases the risk of CRC [3, 4]. However, the relationship between visceral obesity and the prognostic outcome in CRC is inconclusive [4–7]. Visceral obesity is closely related with dysregulated visceral adipose tissue activity [8]. This dysregulated visceral adipose tissue secretes increased adipokines including interleukin-6 (IL-6) and tumor necrosis factor-alpha (TNF-α) [8–10]. These increased adipokines are related with systemic inflammation and can play a role in tumorigenesis and metastasis [8–10]. In addition, elevated inflammatory factors in serum have been significantly associated with metastatic status of CRC patients [11–13]. It is conceivable that increased inflammatory condition of visceral adipose tissue activity might affect the status of regional LN or distant metastasis in CRC patients.

^18^F-fluorodeoxyglucose (FDG) positron emission tomography combined with computed tomography (PET/CT) is an established non-invasive image modality for increased glucose metabolism in inflamed tissue [14, 15]. ^18^F-FDG PET/CT can be used to measure the increased inflammatory condition of visceral adipose tissue activity.

The aim of this study was to investigate the role of functional visceral fat activity assessed by preoperative ^18^F-FDG PET/CT in CRC to predict regional LN or distant metastasis.

## Materials and Methods

### 1. Patients

The retrospective study included 131 patients (79 men and 52 women; mean age, 64±11.6 years) with newly diagnosed CRC from January 2013 to January 2015. They all underwent preoperative ^18^F-FDG PET/CT and surgery. All the specimens were histopathologically confirmed. The patients who received chemotherapy, radiotherapy or stent insertion prior to surgery were excluded. For this type of retrospective study formal consent was not required. Patient records/information was anonymized and de-identified prior to analysis. This study was approved by Korea University Anam Hospital Institutional Review Board (AN15118-01) in accordance with the Declaration of Helsinki of the World Medical Association.

### 2. ^18^F-FDG PET/CT study protocol

Images were acquired with a Gemini TF PET/CT scanner (Philips Medical Systems, Cleveland, OH, USA). All patients fasted for at least 6 h and serum glucose level was <180 mg/dL before scanning. Sixty minutes after intravenous injection of 5.29 MBq/Kg (0.14 mCi/kg) ^18^F-FDG, CT scans were obtained followed by PET emission scans for 1 min. The PET unit had an axial field of view of 18 cm and a spatial resolution of 4.4 mm. A low-dose CT scan was obtained for attenuation correction and for localization, with a 16-slice multidetector helical CT unit, using the following parameters: 120 kVp; 50 mA; 0.75-s rotation time; 0.75-mm slice collimation; 4-mm scan reconstruction, with a reconstruction index of 4 mm; 60-cm field of view; and 512x512 matrix. PET data were reconstructed iteratively using the three-dimensional Row Action Maximum Likelihood Algorithm (RAMLA) with low-dose CT datasets for attenuation correction. Maximum intensity projection (MIP) and cross sectional views and fusion images were generated and reviewed.

### 3. ^18^F-FDG PET/CT image analysis (functional fat activity)

Image analysis was performed in a region of interest (ROI) using the Extended Brilliance Workspace (EBW, Philips Medical Systems) by determining the standardized uptake value (SUV). SUV was calculated as *mean activity (ROI; MBq/g)/ injected dose (MBq)/ total body weight (g)*

Fat areas including visceral fat (VAT) and subcutaneous fat (SAT) were identified by using pre-defined Hounsfield units (HU, range -70 to -110) from background CT images [15]. To measure the VAT activity, ROIs (7-15mm) were located on three consecutive slices of abdominal VAT above or below the kidneys to exclude overspill physiologic F-18 FDG uptake of kidneys, as previously described [15]. In addition, to avoid overspill uptake from primary tumor, vessel, muscle, and/or intestine, ROIs were located at least 2cm away from the previously mentioned structures. The average SUVmax of these three ROIs were acquired. For SAT analysis, three consecutive ROIs were located on the buttock area (postero-lateral aspect of gluteus muscles at iliac wings). Averaged SUVmax of these three ROIs were also acquired. The VAT/SAT (V/S) ratio to measure the functional visceral fat activitywas calculated as follows:

V/S ratio= Averaged VAT SUVmax/ Averaged SAT SUVmax

With these functional parameters, analysis was performed according to metastatic status. Then threshold for discriminating metastatic status was acquired by receiver-operating characteristic (ROC) analysis

### 4. Serum inflammatory marker analysis

We used C-reactive protein (CRP) as a serum inflammatory marker [11–13]. CRP measurement was carried out within 1 month before taking surgery for excluding inflammation associated with surgical procedure. Among 131 patients, 75 patients had CRP results which were collected in this period. None of the patients showed clinical features of acute infection or other acute inflammatory conditions.

### 5. Statistical analysis

The Mann-Whitney *U* test, χ^2^ test, Fisher’s exact test, receiver-operating characteristic (ROC) analysis, Spearman’s correlation coefficient, and uni- and multivariate logistic regression analysis were used as statistical methods. A *p*-value *<*0.05 was defined as statistically significant. SPSS software version 17.0 (SPSS Chicago, IL, USA) and Medcalc software (Medcalc, Mariakerke, Belgium) were used for data analyses.

## Results

Of the 131 patients, 64 were pathologically confirmed as regional LN metastasis (LM), 27 as negative regional LM. 13 as distant metastasis (DisM), and 118 as negative DisM. Among the 13 positive DisM patients, 7 presented positive DisM to liver, 3 to lung, 1 to liver and bone, 1 liver and lung, and 1 to superior mesenteric artery LN. Of the 118 negative DisM patients, 53 patients confirmed as regional LM, and 65 as negative regional LM. The overall characteristics of the patients are shown in Table 1.

**Table 1:** Overall patient characteristics.

	No. Patients	%
No. Patietns	131	
Age >64y		
Yes	69	52.7
No	62	47.3
Sex		
Male	79	60.3
Female	52	39.7
Tumor location		
Colon	88	67.2
Ascending	24	18.3
Transverse	12	9.2
Descending	7	5.3
Sigmoid	45	34.4
Rectum	43	32.8
Differentiation		
Well	24	17.6
Moderate and poor	107	82.4
pT stage		
Tis	1	0.8
T1	5	3.8
T2	15	11.5
T3	96	73.3
T4	14	10.6
N stage		
N0	67	51.1
N1	44	33.6
N2	20	15.3
M stage		
M0	118	90.1
M1	13	9.9
AJCC stage		
0	1	0.8
I	16	12.2
II	48	36.6
III	53	40.5
IV	13	9.9
Lymphatic invasion		
Positive	36	27.5
Negative	95	72.5
Venous invasion		
Positive	5	3.8
Negative	126	96.2
Peineural invasion		
Positive	10	7.6
Negative	121	92.4

pT = Pathologic T stage

AJCC = American Joint Committee on Cancer

### 1. Comparison of functional parameters according to DisM status

**Fig 1:**
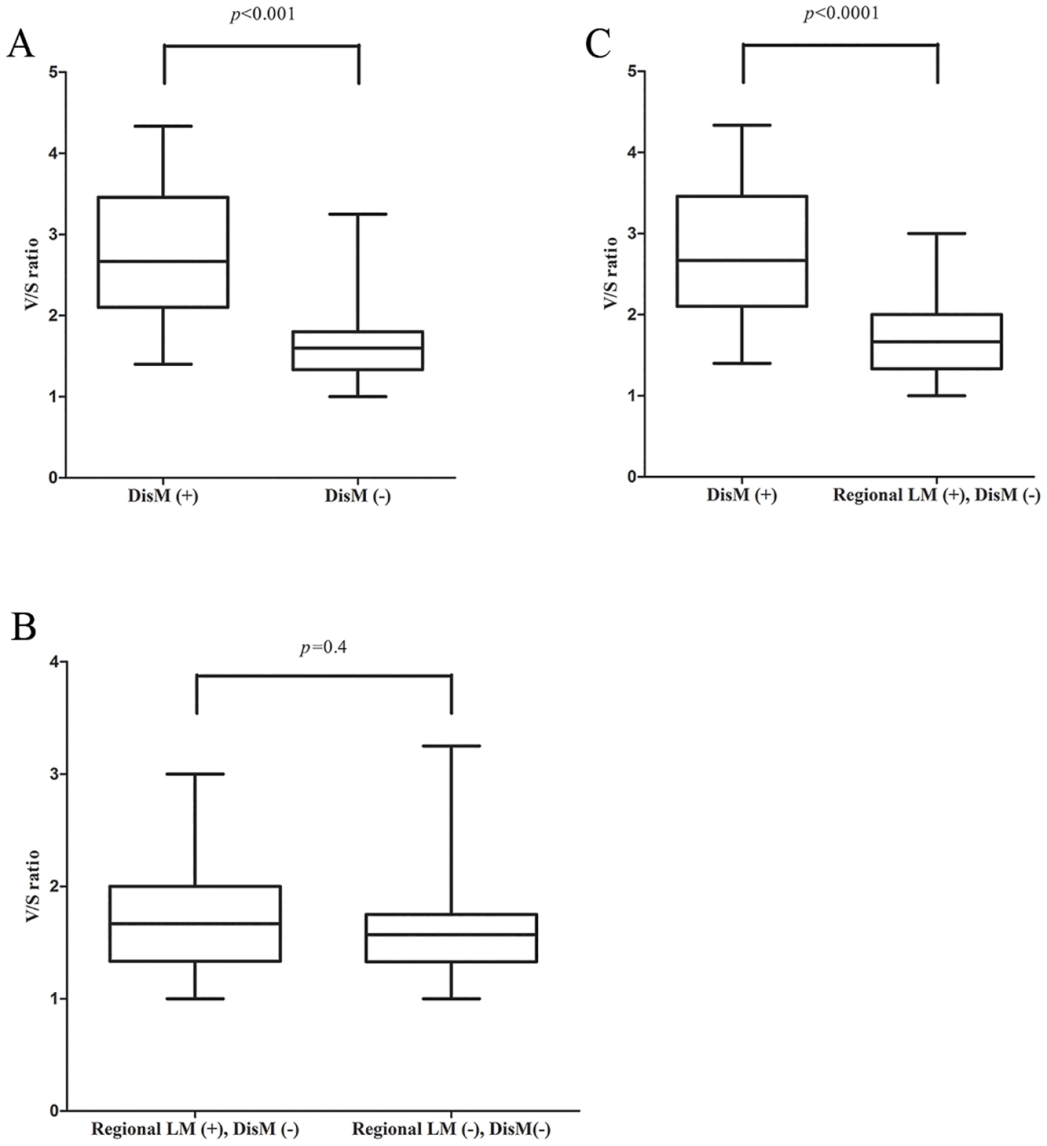
Comparison of averaged visceral fat standardized uptake value/ averaged subcutaneous fat standardized uptake value (V/S) ratio. (A)Between distant metastasis (DisM) and negative DisM. (B)Between regional lymph node metastasis (LM) without DisM and negative regional LM without DisM. (C)Between DisM and regional LM without DisM.

The DisM group showed a significantly higher VAT SUVmax than the negative DisM group (mean ± standard deviation, 1.21±0.39 vs. 0.76±0.27, *p*<0.001). There was no statistically significant difference between the SAT SUVmax of DisM group and the negative DisM group (mean ± standard deviation, 0.45±0.11 vs. 0.47±0.15, *p* = 0.98). The DisM group presented a significantly higher V/S ratio than the negative DisM group (mean ± standard deviation, 2.75±0.88 vs. 1.67±0.47, *p*<0.001; Fig 1A). There was no statistically significant difference between the primary tumor SUVmax of DisM group and the negative DisM group (mean ± standard deviation, 11.64±5.27 vs. 11.37±6.58, *p* = 0.59).

### 2. Comparison of functional parameters according to regional LM status

There was no statistically significant difference between the VAT SUVmax, SAT SUVmax, and V/S ratio of regional LM without DisM group and the negative regional LM without DisM group (mean ± standard deviation, 0.76±0.29 vs. 0.76±0.26, *p* = 0.91, 0.46±0.17 vs. 0.48±0.14, *p* = 0.32, 1.74±0.55 vs. 1.62±0.39,*p* = 0.4; Fig 1B, respectively). There was also no statistically significant difference between the primary tumor SUVmax of regional LM without DisM group and the negative regional LM without DisM group (mean ± standard deviation, 11.02±6.96 vs. 11.66±6.3, *p* = 0.36).

### 3. Comparison of functional parameters between DisM and regional LM without DisM

The DisM group displayed a significantly higher VAT SUVmax and V/S ratio than the regional LM without DisM group (mean ± standard deviation, 1.21±0.39 vs. 0.76±0.29, *p*<0.001, 2.75±0.88 vs. 1.74±0.55, *p*<0.0001; Fig 1C, respectively). There was no statistically significant difference between the SAT SUVmax and the primary tumor SUVmax of DisM group and the regional LM without DisM group (mean ± standard deviation, 0.45±0.11 vs. 0.46±0.17, *p* = 0.79, 11.64±5.27 vs. 11.02±6.96, *p* = 0.41, respectively).

### 4. Determination of cut-off value to discriminate DisM from negative DisM

**Fig 2:**
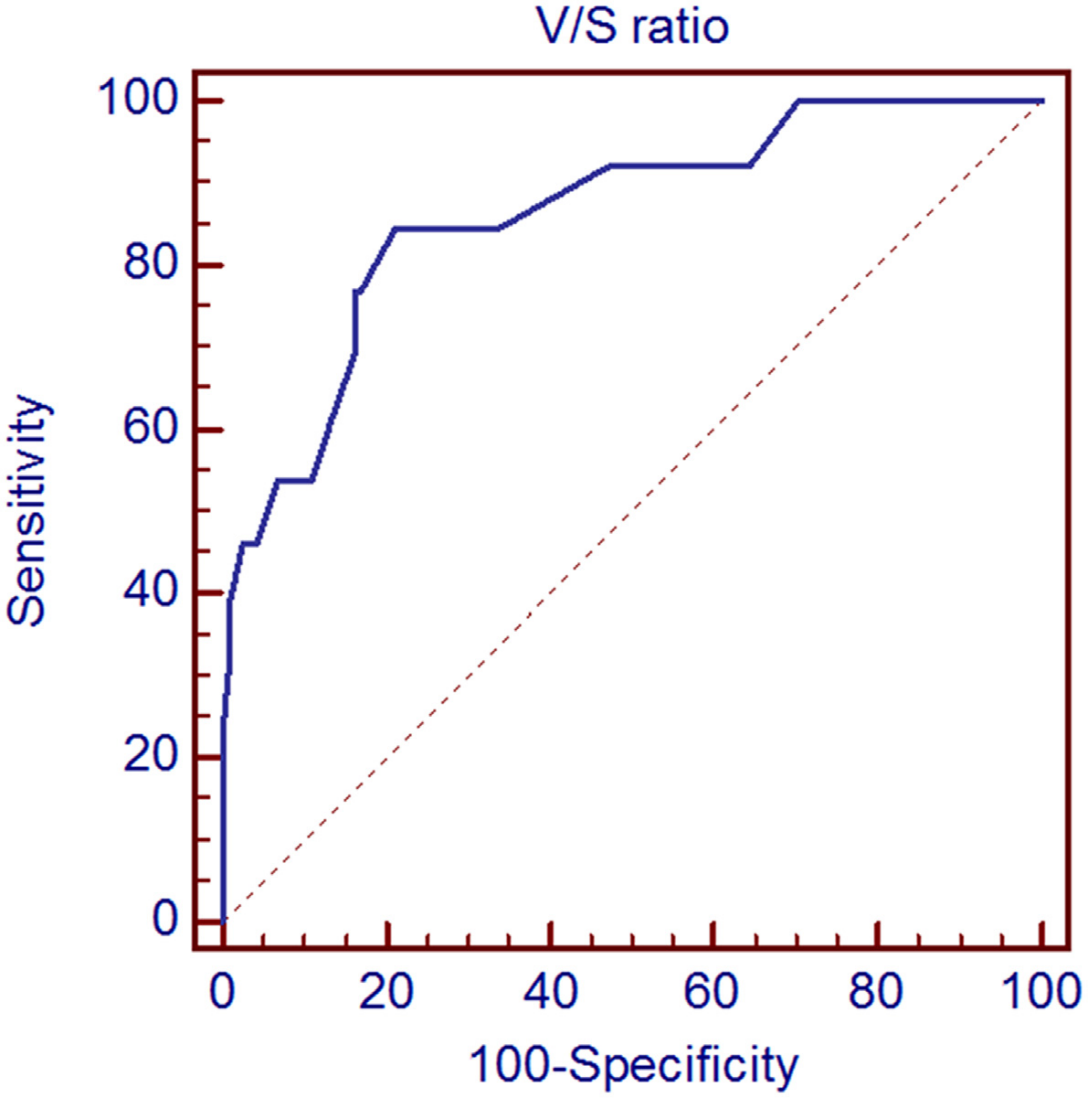
Receiver operating characteristic (ROC) analysis of maximum standardized uptake value of visceral fat/ maximum standardized uptake value of subcutaneous fat (V/S) ratio in colorectal cancer patients for the prediction of distant metastasis.

An optimal cut-off V/S ratio of 1.88 was proposed for prediction of DisM with a sensitivity of 84.6% and specificity of 78.8% (Fig 2). Area under the curve (AUC) was 0.862 (standard error 0.06; 95% confidence interval 0.79–0.92) with a *p*-value of <0.001.

### 5. Comparison of patient groups according to cut-off V/S ratio

Based on the cut-off V/S ratio of 1.88, patients with a higher V/S ratio (>1.88) group displayed a significantly higher rate of lymphatic invasion (*p =* 0.025),regionalLM (*p =* 0.004),DisM (*p<*0.001), and higher American Joint Committee on Cancer (AJCC) stage (*p =* 0.003) (Table 2) than the patients with not exceeding the cut-off value (≤1.88).

**Table 2:** Patient characteristics with functional visceral fat activity.

	V/S ratio>1.88 (36 patients)	V/S ratio≤1.88 (95 patients)	*p*
Age (years)	64±12	64±11	0.173
Sex			0.494
Male	20 (55.6%)	59 (62.1%)	
Female	16 (44.4%)	36 (37.9%)	
Tumor location			0.734
Colon	25 (69.4%)	63 (66.3%)	
Rectum	11 (30.6%)	32 (33.7%)	
Differentiation			0.217
Well	4 (11.1%)	20 (21.1%)	
Moderate and poor	32 (88.9%)	75 (78.9%)	
pT stage			0.186
Tis-T2	3 (8.3%)	18 (18.9%)	
T3-T4	33 (91.7%)	77 (81.1%)	
N stage			0.004*
Negative	11 (30.6%)	56 (58.9%)	
Positive	25 (69.4%)	39 (41.1%)	
M stage			<0.001*
M0	25 (69.4%)	93 (97.9%)	
M1	11 (30.6%)	2 (2.1%)	
AJCC stage			0.003*
0-II	10 (27.8%)	55 (57.9%)	
III-IV	26 (72.2%)	40 (42.1%)	
Lymphatic invasion			0.025*
Negative	21 (58.3%)	74 (77.9%)	
Positive	15 (41.7%)	21 (22.1%)	
Venous invasion			0.615
Negative	34 (94.4%)	92 (96.8%)	
Positive	2 (5.6%)	3 (3.2%)	
Perineural invasion			0.137
Negative	31 (86.1%)	90 (94.7%)	
Positive	5 (13.9%)	5 (5.3%)	

SUVmax = maximum standardized uptake value

V/S ratio = Visceral fat SUVmax/Subcutaneous fat SUVmax ratio

AJCC = American Joint Committee on Cancer

pT stage = Pathologic T stage

* Statistically significant difference

### 6. Uni- and multivariate analyses

Higher pathologic T stage and positive lymphatic invasion were significantly associated with regional LM without DisM by uni- and multivariate analysis (Table 3).

**Table 3:** Uni- and multivariate analyses for predicting regional lymph node metastasis without distant metastasis.

Variables	Univariate analysis			Multivariate
	*RR*	95%CI	*p*	*p*
Age (Continous)	1.006	0.969–1.044	0.764	
Sex (Male vs. Female)	1.008	0.434–2.343	0.985	
Tumor location (Colon vs. Rectum)	1.83	0.696–4.813	0.221	
Differentiation(Well vs. Moderate, poor)	1.08	0.401–2.909	0.879	
pT stage (Tis-T2 vs.T3-T4)	4.714	1.287–17.267	0.019*	0.024*
Lymphatic invasion (Negative vs. Positive)	3.641	1.382–9.593	0.009*	0.004*
Venous invasion (Negative vs. Positive)	0.304	0.026–3.514	0.34	
Perineural invasion (Negative vs. Positive)	1.883	0.258–13.742	0.533	
V/S ratio (≤1.88 vs. >1.88)	1.868	0.682–5.113	0.224	

*pT stage* pathologic T stage, *RR* relative risk, *CI* confidence interval, *SUVmax* maximum standardized uptake value,*V/S ratio*visceral fat SUVmax/subcutaneous fat SUVmax ratio

*p< 0.05 is considered significant

Younger age, positive N stage, positive perineural invasion, higher V/S ratio presented significant association with DisM by univariate analysis (Table 4). In addition, after adjusting these selected factors in a multivariate analysis, higher V/S ratio was the only factor that was significantly associated with DisM (*p =* 0.001).

**Table 4:** Uni- and multivariate analyses for predicting distant metastasis.

Variables	Univariate analysis			Multivariate
	*RR*	95%CI	*p*	*p*
Age (Continous)	0.948	0.902–0.997	0.039*	0.361
Sex (Male vs. Female)	0.648	0.189–2.26	0.491	
Tumor location (Colon vs. Rectum)	0.9	0.261–3.107	0.868	
Differentiation(Well vs. Moderate, poor)	2.23E+08	0.000-.	0.998	
pT stage (Tis-T2 vs.T3-T4)	2.17E+08	0.000-.	0.998	
N stage (Negative vs. Positive)	6.745	1.432–31.77	0.016*	0.164
Lymphatic invasion (Negative vs. Positive)	0.121	0.783–8.073	0.121	
Venous invasion (Negative vs. Positive)	2.375	0.245–23.002	0.455	
Perineural invasion (Negative vs. Positive)	4.757	1.062–21.306	0.041*	0.223
V/S ratio (≤1.88 vs. >1.88)	20.46	4.257–98.344	<0.001*	0.001*

*pT stage* pathologic T stage, *RR* relative risk, *CI* confidence interval, *SUVmax* maximum standardized uptake value,*V/S ratio*visceral fat SUVmax/subcutaneous fat SUVmax ratio

*p< 0.05 is considered significant

### 7. CRP analysis

Of the 75 patietns who had CRP results, 8 confirmed as disM, 67 as negative disM. Of the 67 negative disM patients, 29 confirmed as regional LM and 38 as negative regional LM. The DisM group showed a significantly higher CRP value than the negative DisM group (mean ± standard deviation, 25.73±26.68 mg/L vs. 7.41±10.16 mg/L, *p* = 0.01). There was no statistically significant difference between the CRP value of regional LM without DisM group and the negative regional LM without DisM group (mean ± standard deviation, 6.2±8.72 mg/L vs. 8.33±11.16 mg/L, *p* = 0.28). The DisM group displayed a significantly higher CRP value than the regional LM without DisM group (mean ± standard deviation 25.73±26.68 mg/L vs. 6.2±8.72 mg/L, *p* = 0.006). However, we couldn’t find the significant linear correlation between CRP value and V/S ratio (*r* = 0.09, *p* = 0.42).

## Discussion

In this study, higher functional VAT activity defined as V/S ratio determined by preoperative F-18 FDG PET/CT showed a significant higher rate of regional LM and DisM in CRC patients. In addition, DisM group showed significantly higher V/S ratio than negative DisM group. Christen *et al*. [16] reports that VAT SUVmax is higher than SAT SUVmax in normal population and the normal ranges of VAT SUVmax and SAT SUVmax are 0.81±0.23 to 0.88±0.18 vs. 0.30±0.09 to 0.33±0.08, respectively. Our results were concordant with this previous report and DisM group presented higher VAT SUVmax and V/S ratio than normal population group.

Through CRP analysis, DisM group showed significantly higher CRP values than negative DisM group. Our results were concordant with previous study [12]. Therefore, it is possible to say that DisM group is more inflamed than negative DisM group.

Macrophages, especially M1, play a major role in increased inflammatory response in VAT [17]. M1 macrophages secrete proinflammatory cytokines including TNF-α, IL-6, IL-8, IL-12, and IL-23, and produce high levels of oxygen radicals and superoxide anions [17]. These activated M1 macrophages can enhance tumor aggressiveness [17]. Interestingly, F-18 FDG uptake is greatest in M1 macrophages and increased F-18 FDG uptake might indicate increased M1 macrophage activity [14].

Our findings support a mechanism in which increased inflammatory response by increased functional VAT activity can affect the metastatic status in CRC patients [10].

We found that a higher V/S ratio showed significant association with DisM than regional LM without DisM. In addition, it was a useful factor in predicting DisM. That is to say, higher V/S ratio significantly affects the status of DisM than regional LM only. This result may reflect the different level of activated inflammatory response between DisM and regional LM without DisM. Presently, patients with DisM displayed a significantly higher V/S ratio than the regional LM without DisM group (Fig 1C). CRP levels were also significantly higher in DisM than the regional LM without DisM group. These observations combined with the prior finding that DisM group shows higher IL-6 and IL-8 than CRC patients with regional LM only group [18] indicate that DisM might be associated with a greater inflammatory response than regional LM only.

Several previous studies reported the relationship between visceral obesity and the prognosis of CRC [5–7]. However, the results were diverse and discordant. These studies used CT to measure VAT volume as a surrogate marker of VAT activity. However, VAT volume is reportedly unrelated to visceral fat inflammation [19] and determination of VAT volume by CT may not be sufficient to reflect the actual functional VAT activity [15, 16, 19]. Therefore, a functional imaging modality like F-18 FDG PET/CT could be more suitable for assessment of functional VAT activity than CT.

Previous studies regarding functional visceral fat activity and F-18 FDG PET/CT focused on non-oncologic systemic inflammatory diseases, such as atherosclerosis or chronic obstructive pulmonary disease [15, 20, 21]. The present study used F-18 FDG PET/CT to demonstrate the oncologic application of functional visceral fat activity, which can provide molecular information about inflammatory processes in CRC metastasis.

Our study has several limitations. It was retrospective and was conducted on relatively small population in a single center. Further prospective studies with larger populations will be necessary to validate our results.

Despite these limitations, this study clearly identifies that functional visceral fat activity assessed by F-18 FDG PET/CT can predict the status of DisM in CRC patients.

In conclusion, functional visceral fat activity assessed by F-18 FDG PET/CT is significantly associated with DisM. Furthermore, it is a useful factor for the prediction of DisM in CRC patients.
